# The Combination of Host-Associated *Bacillus megaterium* R32 and Stachyose Promotes the Intestinal Health of Turbot (*Scophthalmus maximus*. L)

**DOI:** 10.1155/2024/8658386

**Published:** 2024-09-30

**Authors:** Yaoyao Kong, Sifan Zhao, Weihao Ou, Kangsen Mai, Yanjiao Zhang

**Affiliations:** ^1^ The Key Laboratory of Aquaculture Nutrition and Feed (Ministry of Agriculture) and the Key Laboratory of Mariculture (Ministry of Education) Ocean University of China, 5 Yushan Road, Qingdao, Shandong 266003, China; ^2^ Laboratory for Marine Fisheries Science and Food Production Processes Qingdao National Laboratory for Marine Science and Technology, 1 Wenhai Road, Qingdao, Shandong 266237, China

**Keywords:** *Bacillus megaterium* R32, intestinal barriers, intestinal immunity, *Scophthalmus maximus*. L, stachyose

## Abstract

An 8-week feeding trial was conducted to investigate the effects of host-associated *Bacillus megaterium* R32 and stachyose on the intestinal mucosal defense system of turbot (*Scophthalmus maximus*. L). Three isonitrogenous and isolipidic diets were formulated: control diet (C), control diet with 1.0 × 10^8^ CFU/g *B. megaterium* R32 (RC), and 1.0 × 10^8^ CFU/g *B. megaterium* R32 + 1.5% stachyose (RS) supplementation separately. The results showed that diets RS and RC significantly inhibited the expression of cell development and apoptosis-related genes (*β-catenin*, *CyclinD1*, *BAX*, *Bid*); diets RS and RC significantly increased the expression of intestinal tight junction protein *claudin-4*, while RS group significantly decreased the expression of *myosin light chain kinase*; diets RS and RC significantly decreased the expression of proinflammatory factors (*IL-13*, *IL-15*, *IFN- γ*), diet RS also significantly decreased the expression of *TNF-α* and *AP-1*, and increased the expression of *TGF-β*. 16s rRNA gene sequencing results showed that diets RS and RC significantly decreased the abundance of conditional pathogenic bacteria (*Corynebacterium*, *Desulfovibrio*, *Escherichia-Shigella*). Among them, the abundance of *Bacillus* in the RS group was the highest. It is concluded that the combination of stachyose and *B. megaterium* R32 had a more positive effect on intestinal cell development and barrier function and strengthened the intestinal mucosal defense system of turbot.

## 1. Introduction

Disease is a key factor restricting the development of aquaculture, and the addition of probiotics to feed is considered to be an important way to improve the disease resistance of aquatic animals. *Bacillus* has been widely reported as probiotics in tilapia (*Oreochromis niloticus*), whiteleg shrimp (*Penaeus vannamei*), triangular bream (*Megalobrama terminalis*), olive flounder (*Paralichthys olivaceus*), seabass (*Dicentrarchus labrax*), and rainbow trout (*Oncorhynchus mykiss*) because of its excellent probiotic properties, such as inhibiting the proliferation of pathogenic bacteria, secreting digestive enzymes, immune stimulation, and regulating intestinal microecology [1–6]. In addition, studies have shown that, compared with the addition of exogenous probiotics, host-associated probiotics are weakly attacked by host immune cells, have stronger host specificity and adaptability, and can play a better role in probiotics [7]. Compared with only adding probiotics or adding prebiotics at the same time, the addition of prebiotics is more beneficial to the colonization of probiotics and improves the immune function of the host [8, 9]. As a kind of prebiotics, stachyose can regulate immunity, relieve intestinal inflammation, enhance digestion and absorption, and improve growth performance [10–13]. It has been shown that the appropriate addition of stachyose can increase the abundance of *Bacillus* in the intestine of turbot (*Scophthalmus maximus*. L) [14].

As the largest immune organ, the intestine plays an important role in establishing immunity and eliminating endotoxin and is the first line of defense against pathogen invasion [15]. Intestinal epithelial cells are closely arranged through cell junctions, in which cell junctions are mainly tight junctions, which can effectively block the entry of bacteria, viruses, and endotoxins. Once the tight junctions of intestinal epithelial cells are mutated, reduced, or missing, the interstitial permeability of intestinal epithelial cells will increase, and bacteria, endotoxins, and macromolecules can enter the systemic circulation through the tight junctions [16]. The intestinal mucosal immune system consists of a unique series of congenital and acquired immune cells and molecules that work together to protect the host from pathogens. Immune cells can maintain immune balance by regulating the secretion of inflammatory factors or secreting immunoglobulin to block the adhesion of foreign pathogens and other antigens to the mucosa [17]. In addition, probiotics in the intestine can also prevent the proliferation of pathogenic bacteria by secreting bacteriocin and competing for ecological sites [18].

Turbot is one of the important economic fish in mariculture, which has the characteristics of high nutritional value and delicious meat quality. However, the high density of cultivation makes turbot disease frequent. *Bacillus megaterium* R32, a strain of bacteria isolated from the intestine of a healthy turbot, was first found and identified. Thus, the present study aimed to evaluate the effect of host-associated probiotics (*B. megaterium* R32), as well as the combination of *B. megaterium* R32 and stachyose on intestinal health of turbot, so as to provide theoretical support for the development of microecological agents with immunomodulatory activity.

## 2. Materials and Methods

### 2.1. Ethics Statement

All experimental protocols involved in this study were approved by the Ocean University of China Academic Committee (ethics approval number: OUC-AE-2024-201).

### 2.2. Isolation, Identification, and Culture of *B. megaterium*

Strain R32 was obtained from the intestines of a healthy turbot. The neighbor-joining phylogenetic tree based on 16s rRNA gene sequence alignment of strain R32 found that strain R32 was closest to *B. megaterium* NBRC 15308. Strain R32 stored at −80°C was thawed and cultured in a medium to obtain suspension for feed production. The formulation of the spore solid medium is shown in Table 1.

### 2.3. Experimental Diets

Three experimental diets were prepared in this experiment. The experimental diet formulations are listed in Table 2. The raw materials were added according to the principle of step-by-step amplification and then gradually mixed. Fish oil and water were added to the mixture twice in proportion, kneaded evenly, and passed through an 80-mesh sieve. The sieved raw materials were put into the blender and mixed thoroughly before granulation. The pelleted feed obtained was sprayed with the same amount of sterile water or bacterial solution according to the treatment and then dried in an oven at 50°C and stored at −20°C.

### 2.4. Rearing Conditions

Juvenile turbots were purchased from Yantai Development Zone Tianyuan Aquatic Products Co., Ltd. The turbot-rearing experiment was also carried out in the flowing water system. Before the start of rearing, the turbots were fed with a control diet for 2 weeks to adapt to the rearing environment. After the temporary rearing, 270 fish were randomly distributed into 9 buckets, with 30 fish in each bucket and 3 replicates for each treatment. Turbots were fed twice a day (7:00 and 16:00) to satiation. During this period, the water temperature was 17–20°C, the dissolved oxygen was 6–8 mg/l, the salinity was 27−29‰ and the pH was 7–8.

### 2.5. Sampling

After the 8-week of feeding, all fish were starved for 24 h. Ten fish were randomly selected from each bucket and anesthetized with eugenol (1:10,000) (> 99%, Shanghai Reagent Company, China). The hindgut was removed with sterilized scissors and forceps and immediately frozen in liquid nitrogen. After the experiment, the turbot was packed in plastic bags and placed in a specially marked freezer to be collected by the school's Equipment and Laboratory Management Office for unified disposal. After sampling, all samples were transferred to −80°C for subsequent analysis. Six fish were randomly selected from the RC treatment group, and the midgut, midgut contents, hindgut, and hindgut contents were collected in sterile 2 ml centrifuge tubes (sterile grinding beads and 1 ml sterile PBS were added to the 2 ml centrifuge tubes in advance and weighed) and stored at 4°C.

### 2.6. Survival Analysis of *B. megaterium* R32

After weighing, the intestinal samples of the RC group were prepared into homogenate, diluted 10 times according to gradient, evenly spread on TSA medium, and cultured at 28°C for 24 h. After the culture, each plate was counted. Three colonies were randomly selected for DNA extraction and PCR reaction. The PCR products were sequenced by Tianyi Huiyuan Biotechnology Co., Ltd. (Beijing, China) and confirmed according to the obtained sequences.

### 2.7. Intestinal RNA Extraction, cDNA Synthesis, and Quantitative Real-Time PCR

The total RNA of the samples in this experiment was extracted by using RNAex Pro Reagent (AG21102, Accurate Biotechnology [Hunan] Co., Ltd., Changsha, China). By analyzing the results of 260/280 and gel electrophoresis to ensure that the quality of RNA meets the requirements of follow-up experiments. RNA was reverse transcribed into cDNA by Evo M-MV RT Mix Kit with gDNA Clean for qPCR Ver.2 (AG11728, Accurate Biotechnology [Hunan] Co., Ltd., Changsha, China). *β*-Actin was selected as the internal reference gene in this experiment. The primers of all genes were designed on NCBI and synthesized in Sangon Biotech (Shanghai) Co., Ltd. The details are listed in Table 3.

### 2.8. DNA Extraction and High-Throughput Sequencing of Intestinal Microbiota

In this experiment, the genome of intestinal mucosal microbiota was extracted by QIAamp PowerFecal Pro DNA Kit. All operating procedures shall be subject to the instructions. The brief description is as follows: the hindgut mucosa samples were centrifuged with CD1, CD2, and CD3 successively, the supernatant was absorbed and added with the same amount of lysate, then EA, C5, and C6 were added successively, and centrifuged, and finally the supernatant was discarded to get DNA.

In order to analyze the differences in microbial composition among 15 intestinal samples, 16s rDNA analysis was carried out on the V4 region of the samples. Using sample DNA as a template, 16s rRNA gene V4 region primers (515F and 806R) with barcodes were used for PCR, and PCR products were recovered. The library was constructed by the TruSeq DNA PCR-Free Sample Preparation Kit library kit, and the constructed library was quantified by Qubit and Q-PCR. The qualified library is used for subsequent computer sequencing. The sequencing strategy and platform selected were PE250 and Illumina novaseq, respectively. All the sequencing processes were carried out in Tianjin Novogene Genomics Technology Co. Ltd. (Tianjin, China).

### 2.9. Sequencing Data Analysis of Intestinal Microbiota

Bioinformatic analysis of the gut microbiota was carried out using the Majorbio Cloud platform (https://cloud.majorbio.com). Alpha diversity indices were calculated with Mothur v1.30.1. The similarity among the microbial communities in different samples was determined by principal coordinate analysis (PCoA) based on Bray–Curtis dissimilarity using the Vegan v2.5-3 package.

### 2.10. Calculations

The following variables were calculated:

  $$\begin{array}{c} \text{Specific growth rate }\left(\text{SGR},\%\text{ day}\right)=100\times \left(\text{Ln final weight}-\text{ Ln initial weight}\right)/\text{days} \end{array}$$

  $$\begin{array}{c} \text{Feed efficiency }\left(\text{FE}\right)=\left(\text{final weight}-\text{ initial weight}\right)/\text{feed consumed} \end{array}$$

  $$\begin{array}{c} \text{Feed rate }\left(\text{FR},\%/\text{day}\right)=100\times \text{feed intake}\left[\left(\text{initial weight}+\text{ final weight}\right)/2\right]/\text{days} \end{array}$$

  $$\begin{array}{c} \text{Survival rate }\left(\text{SR},\%\right)=100\times \left(\text{final amount}/\text{initial amount}\right) \end{array}$$

### 2.11. Statistical Analysis

All statistical analyses except for microbiota composition were performed using SPSS for Windows (V 22.0, IBM, US). Data were subjected to one-way ANOVA followed by Turkey's HSD test. Wilcoxon test was carried out when needed. Comparisons of two means were performed by using a two-tailed Student's *t*-test. Data were expressed as means ± standard of the means.

## 3. Results

### 3.1. Specific Growth Rate, Feeding Rate, Feed Efficiency Rate, and Survival Rate of the Turbots

The results of the specific growth rate, feeding rate, and feed efficiency rate of turbots are shown in Table 4. Compared with the control group, there were no significant differences among the groups.

### 3.2. The Survival of *B. megaterium* R32 on Intestine and Intestinal Contents of Turbot

**Figure 1:**
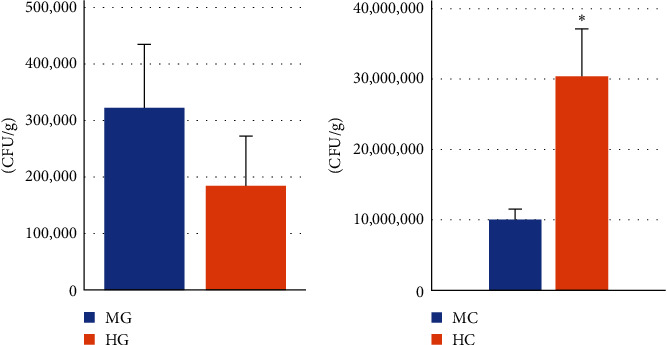
The survival of *B. megaterium* R32 on intestine (A) and intestinal contents (B) of turbot from the RC group. MG means midgut; HG means hindgut; MC means midgut contents; HC means hindgut contents. “ ^*∗*^” Means significant difference (*P* < 0.05).

As shown in Figure 1, the amount of *B. megaterium* R32 in the midgut and hindgut mucosa were 3.22 × 10^5^ and 1.85 × 10^5^ CFU/g, respectively, with no significant difference. The amount of *B. megaterium* R32 in the content of the hindgut (3.04 × 10^7^) was significantly higher than that in the content of the midgut (1.00 × 10^7^).

### 3.3. Intestinal Development of the Turbot

**Figure 2:**
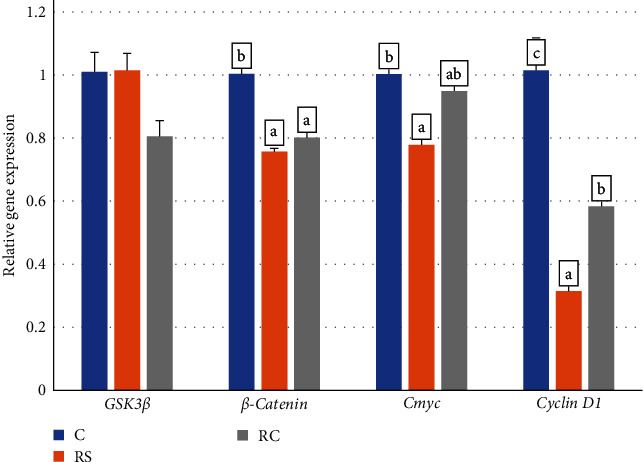
Effects of dietary stachyose and *Bacillus* on the expression of intestinal *Wnt/β-catenin*-related genes (*GSK3β*, *β-catenin*, *Cmyc*, *Cyclin D1*) in turbot. Values were means of triplicate tanks with standard errors. Bars not sharing the same letters were significantly different (*P* < 0.05).

As shown in Figure 2, compared with the control group, the expressions of *CyclinD1* and *β-catenin* were significantly decreased in the RS and RC groups, and the expression of *Cmyc* was significantly decreased in the RS group (*P* < 0.05).

### 3.4. Apoptosis-Related Genes in the Turbot Gut

**Figure 3:**
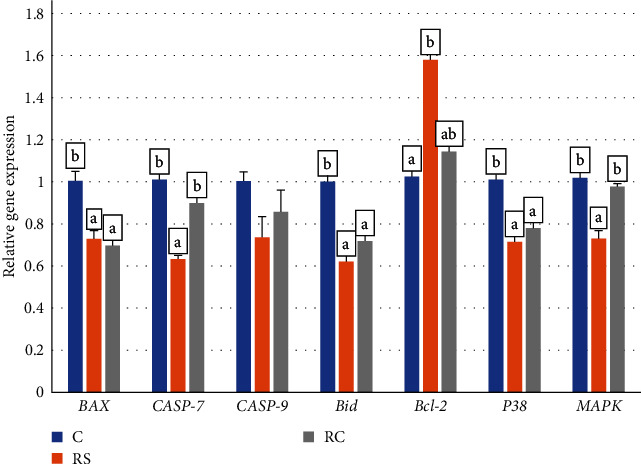
Effects of dietary stachyose and *Bacillus* on the expression of intestinal apoptosis-related genes (*BAX*, *Caspase-7*, *Caspase-9*, *Bid*, *Bcl-2*, *P38*, *MAPK*) in turbot. Values were means of triplicate tanks with standard errors. Bars not sharing the same letters were significantly different (*P* < 0.05).

As shown in Figure 3, compared with the control group, the expressions of *Bax*, *Bid*, and *P38* were significantly decreased in the RS and RC groups (*P* < 0.05). The expression of *Bcl-2* was significantly increased in the RS group, while the expressions of *MAPK* and *Caspase7* were significantly decreased (*P* < 0.05).

### 3.5. Mechanical Barrier in the Turbot Gut

**Figure 4:**
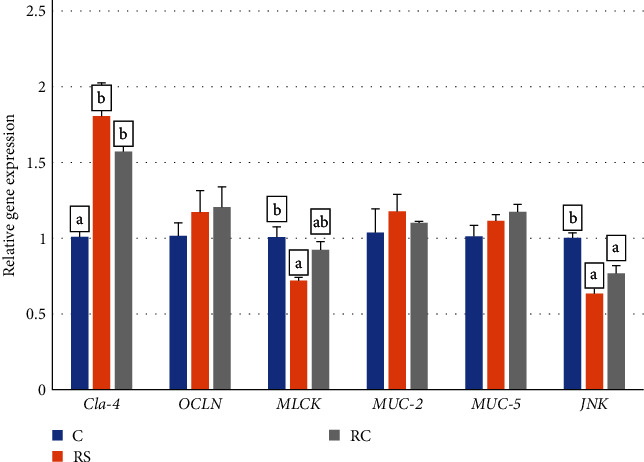
Effects of dietary stachyose and *Bacillus* on the expression of intestinal barrier-related genes (*Claudin4*, *Occludin*, *MLCK*, *Mucin-2*, *Mucin-5*, *JNK*) in turbot. Values were means of triplicate tanks with standard errors. Bars not sharing the same letters were significantly different (*P* < 0.05).

As shown in Figure 4, compared with the control group, the expression of *Claudin-4* was significantly increased in the RS and RC groups (*P* < 0.05). The expression of *JNK* was significantly decreased in all treatment groups, and the expression of myosin light chain kinase (*MLCK*) was significantly decreased in the RS group (*P* < 0.05).

### 3.6. Immune Barrier in the Turbot Gut

**Figure 5:**
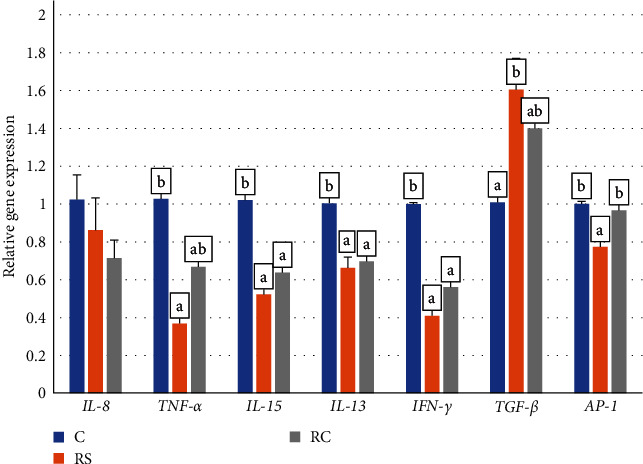
Effects of dietary stachyose and *Bacillus* on the expression of intestinal immune-related genes (*IL-8*, *TNF-α*, *IL-15*, *IL-13*, *IFN-γ*, *TGF-β*, *AP-1*) in turbot. Values were means of triplicate tanks with standard errors. Bars not sharing the same letters were significantly different (*P* < 0.05).

As shown in Figure 5, compared with the control group, the expression of proinflammatory factors *IL-13*, *IL-15*, and *IFN-γ* were significantly decreased in all treatment groups (*P* < 0.05), the expression of *TNF-α* and *AP-1* were significantly decreased in RS group (*P* < 0.05). The expression of *TGF-β* was significantly increased in the RS group (*P* < 0.05).

### 3.7. Composition of the Intestinal Microbiota of Turbot

**Figure 6:**
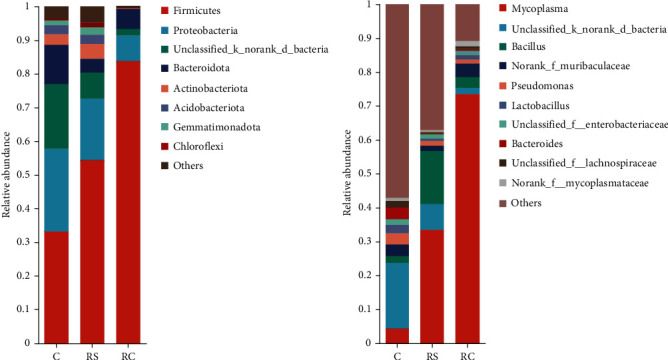
Taxonomy classification of reads at the phylum (A) and genus (B) taxonomic level. Only the top 10 most abundant (based on relative abundance) bacterial phyla and genera were shown in the figures.

As shown in Figure 6, at the phylum level, the dominant phyla of the C, RS, and RC groups were Firmicutes. At the genus level, the dominant genera in all groups were *Mycoplasma*. Among them, the abundance of *Bacillus* in descending order was RS, RC, and control group.

### 3.8. Diversity Analysis of Intestinal Microbiota in Turbot

**Figure 7:**
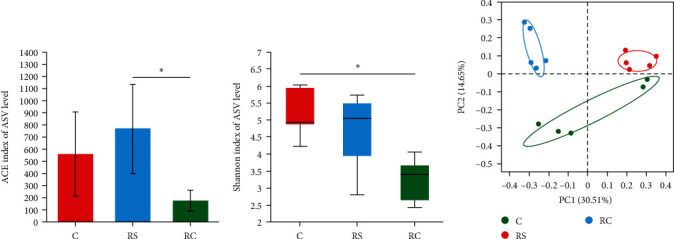
Alpha ((A) ACE; (B) Shannon index) and beta diversity ((C) PCoA) of intestinal microbiota of turbot. Principal coordinate analysis (PCoA) of dissimilarity between bacterial communities based on Unweighted UniFrac distances. “ ^*∗*^” Means significant difference (*P* < 0.05).

As shown in Figure 7, the results of *α*-diversity analysis showed that the Shannon index of the RC group was significantly lower than that of the control group. The analysis of PCoA results showed that the control group and the treatment group were separated on two sides. Although the RS and RC groups were on the same side, they were also in two quadrants, respectively, and the sample clustering of the RS group was the best among the three groups. As shown in Table 5, the Adonis test confirmed that the clustering differences among the groups were extremely significant (*P* < 0.01).

### 3.9. Differential Bacteria Among Different Treatment Groups

**Figure 8:**
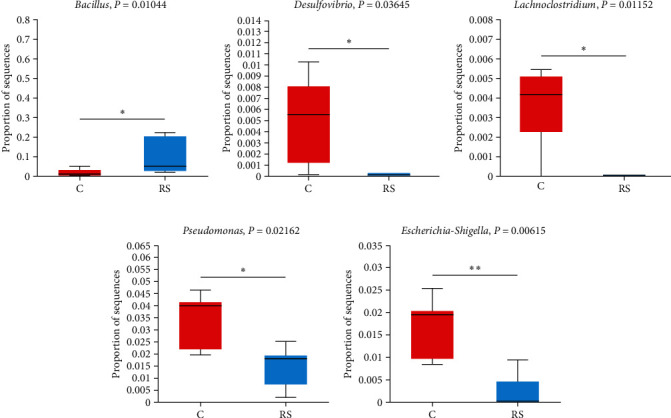
The relative abundance of *Bacillus* (A), *Desulfovibrio* (B), *Lachnoclostridium* (C), *Pseudomonas* (D), and *Escherichia-Shigella* (E) in C and RS groups.“ ^*∗*^” Means significant difference (*P* < 0.05), and “ ^*∗∗*^” means extremely significant difference (*P* < 0.01).

**Figure 9:**
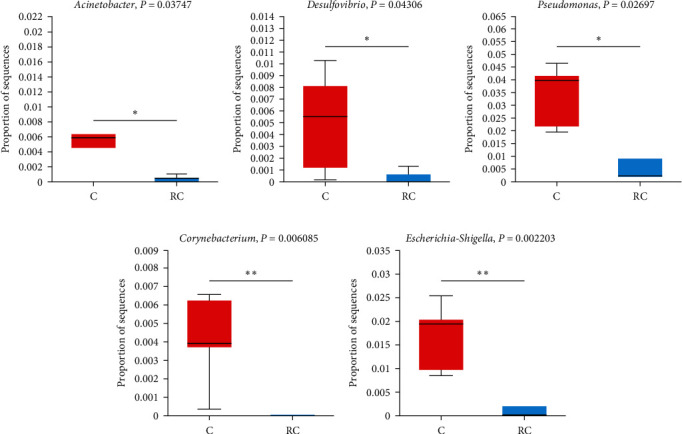
The relative abundance of *Acinetobacter* (A), *Desulfovibrio* (B), *Pseudomonas* (C) and *Corynebacterium* (D), and *Escherichia-Shigella* (E) in C and RC groups.“ ^*∗*^” Means significant difference (*P* < 0.05), and “ ^*∗∗*^” means extremely significant difference (*P* < 0.01).

**Figure 10:**
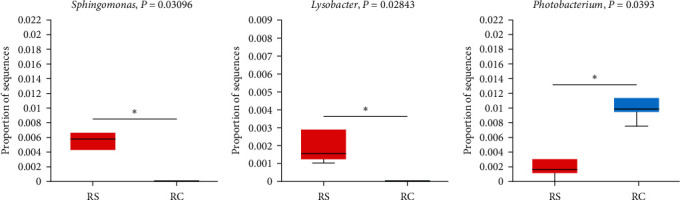
The relative abundance of *Sphingomonas* (A), *Lysobacter* (B), and *Photobacterium* (C) in S and RS groups. “ ^*∗*^” Means significant difference (*P* < 0.05).

A comparative analysis was conducted between the control group and the RS group (Figure 8). The abundance of *Desulfovibrio*, *Escherichia-Shigella*, *Lachnoclostridium*, and *Pseudomonas* in the RS group was significantly lower than that in the control group. The control group was compared with the RC group (Figure 9). The abundance of *Acinetobacter*, *Corynebacterium*, *Desulfovibrio*, *Escherichia-Shigella*, and *Pseudomonas* in the RC group was significantly lower than that of the control group. The RS and RC groups were compared and analyzed, and the abundance of *Sphingomonas* and *Lysobacter* in the RS group was significantly higher than that in the RC group, while the abundance of *Photobacterium* in the RS group was significantly lower than that in the RC group (Figure 10).

## 4. Discussion

There was no significant difference in growth performance between treatment groups, which may be related to the short experimental time, which caused the probiotic effects of *B. megaterium* R32 and stachyose to be more reflected in intestinal health.

Intestinal epithelial cells are arranged closely with each other to form a physical barrier, which becomes the first line of defense of the intestinal tract against pathogenic microorganisms [15]. Therefore, the normal development of intestinal epithelial cells is an important guarantee to prevent microorganisms and toxins from invading the intestine. The results showed that both diet RS and RC could significantly reduce the expression of *β-catenin* and *CyclinD1*, while *Cmyc* was only significantly inhibited when stachyose and *B. megaterium* R32 were combined. Previous studies have confirmed that excessive *β-catenin* content in cells can activate the overexpression of *Cmyc* and *CyclinD1* (downstream genes of the *Wnt/β-catenin* signaling pathway), and the overexpression of these two genes is closely related to abnormal cell proliferation and development [19–27]. Therefore, the combination of stachyose and *B. megaterium* R32 could not only inhibit the activation of the *Wnt/β-catenin* signal pathway but also regulate the expression of *Cmyc* to ensure the normal development of intestinal cells.

Pathogens can not only induce excessive apoptosis to cause injury but also interfere with the apoptosis pathway to achieve immune escape [28]. The addition of probiotics can inhibit apoptosis and reduce the injury of intestinal epithelial cells [29, 30]. This study found that the combination of stachyose and *B. megaterium* R32 can significantly reduce the expression of proapoptotic factors. These results are similar to those obtained in other studies. *B. subtilis* supplemented with 1 × 10^8^ CFU/ml can downregulate the expression of apoptosis genes (*caspase-2*, *caspase-8*, *caspase-9*) in grass carp (*Ctenopharyngodon idella*) [31]. Administration of *Bacillus* to *Penaeus chinensis* can block WSSV infection by inhibiting caspase-3 activity [32]. Yan et al. [33] reported that soluble proteins of probiotics can contact intestinal cells to activate *Akt* and inhibit the *p38/MAPK* signaling pathway to prevent cytokine-induced intestinal epithelial cell apoptosis. Among them, *Akt* can inhibit apoptosis by inactivating several proapoptotic pathways, including *Bax* and *caspase-9* [34]. From the above results, the combination of stachyose and *B. megaterium* R32 may inhibit apoptosis and reduce the injury of intestinal epithelial cells through the *p38/MAPK* signal pathway, but the specific mechanism needs to be further explored.

The complete connection between intestinal epithelial cells constitutes the structural basis of the physical barrier, and the tight junction is the most important connection mode in the structural basis [35]. The results showed that the addition of *B. megaterium* R32 significantly increased the expression of *Claudin-4*. As a pore-blocking protein, the increase of *Claudin-4* expression is beneficial to maintain the stability of intestinal permeability and ensure the balance of substance exchange inside and outside the intestine [36]. In addition, diet RS significantly downregulated the expression of MLCK. It has been reported that the upregulation of *MLCK* will cause the contraction of actin–myosin filaments and lead to the internalization of tight junctions, thus destroying the tight junction structure [37]. Similar results were also reported in studies of Crucian carp (*Carassius auratus gibelio*), Amur minnow (*Rhynchocypris lagowskii*), grass carp, turbot, and gilthead sea bream (*Sparus aurata*) [31, 38–41]. The above results suggested that stachyose and *B. megaterium* R32 can improve the tight junction structure by regulating the expression of tight junction protein and *MLCK*, maintain the stability of intestinal permeability.

The mucosal immune system exists independently from the systemic immune system and is the main barrier against the invasion of pathogenic microorganisms from the mucosa [42]. Nguyen et al. [43] added *Bacillus* to the feed of Channel Catfish (*Ictalurus punctatus*); compared with the control group, the expression of proinflammatory factors in the spleen was significantly decreased. Similarly, Priya et al. [44] indicated that *B. subtilis* can enhance the immune defense activity of Zebrafish (*Danio rerio*). In this study, the expression of proinflammatory factors can be significantly reduced after adding *B. megaterium* R32, and the effect is more significant when combined with stachyose. This may be related to the fact that stachyose itself, as a polysaccharide substance, and the antigenic fragments carried by probiotics, such as polysaccharide cell walls, can pass through intestinal cells and enter the mucosal layer to interact with immune cells [45]. Among them, cytokines are the key regulators that mediate the inflammatory response of fish. The present results showed that the combination of stachyose and *B. megaterium* R32 can maintain intestinal immune homeostasis by balancing the expression of inflammatory factors.

Proteobacteria and Firmicutes were dominant in the control group at the phylum level, which was consistent with the previous studies on the intestinal microbial composition of turbot [46]. At the genus level, *Mycoplasma* was the dominant genus in both RS and RC groups. Many studies have shown that *Mycoplasma* widely exists in the intestinal tract of marine fish. Although there are no known characteristics that are beneficial to the host, there is no evidence to prove its harmful or pathogenic effects [47, 48]. The dominant genus of the RS group was *Bacillus*, and the abundance of *Bacillus* in RC and C groups decreased in turn, indicating that the Stachyose can increase the abundance of *Bacillus* in the intestine of turbot. Due to the lack of *α*-galactosidase in fish, stachyose is difficult to be digested and utilized directly by fish, but it is beneficial to the synthesis of various vitamins and the absorption of trace elements such as calcium and magnesium, thus providing nutrients for the proliferation of probiotics. In addition, short-chain fatty acids such as acetic acid and propionic acid produced by stachyose fermented by intestinal microorganisms can reduce intestinal pH, inhibit the proliferation of pathogens, and increase the ecological occupation of beneficial bacteria [49–53].

The abundance of *Desulfovibrio*, *Escherichia-Shigella*, *Lachnoclostridium*, and *Pseudomonas* in the RS group was significantly lower than that in the control group. *Desulfovibrio* is a kind of anaerobic bacteria that can produce hydrogen sulfide. Endogenous hydrogen sulfide can poison intestinal epithelial cells and cause intestinal sensitivity. It has been confirmed that this bacterium may be involved in the development of ulcerative colitis [54, 55]. *Escherichia-Shigella* belongs to Proteobacteria, which not only can induce the synthesis of LPS, but also the ethanol produced can damage the intestinal mucosa and cause intestinal inflammation [56]. *Lachnoclostridium* has been found to be associated with enteritis, hepatic steatosis, and metabolic diseases in some studies [57]. *Pseudomonas* is also a common conditional pathogen, generally a secondary infection commonly seen in wound infection [58, 59]. The abundance of *Acinetobacter* and *Corynebacterium* was also significantly decreased in the RC group. Most of *Corynebacterium* are conditional pathogens, which are easy to cause superficial and invasive infections and are often isolated as contaminants in clinical samples [60]. As a pathogen, *Acinetobacter* has also been reported in whiteleg shrimp, rainbow trout, and Indian major carp (*Labeo rohita*) [61–63]. Through the comparative analysis of RS and RC groups, the RC group significantly decreased the abundance of *Sphingomonas* and *Lysobacter* and increased the abundance of *Photobacterium*. *Sphingomonas* can degrade aromatic compounds and produce oligosaccharides from alginate by synthesizing alginate lyase [64]. *Lysobacter* has a strong bacteriostatic and antibacterial effect and has strong antagonistic activity against a variety of pathogens [65]. *Photobacterium* is a common marine fish pathogen that can cause skin ulcers, tissue lesions, and other diseases [66]. The microbial results showed that the addition of Stachyose and *B. megaterium* R32 could play a positive role in intestinal flora. Stachyose could help *B. megaterium* R32 proliferate better in the host and balance intestinal homeostasis.

## 5. Conclusions

This study showed that the addition of host-associated *B. megaterium* R32 in feed can strengthen the defense ability of turbot's intestinal tract by improving the tight junction, balancing inflammatory response, maintaining the stable development of intestinal epithelial cells, and improving the communities of intestinal microbiota. Among them, the effect of the combination of *B. megaterium* R32 and stachyose is more positive, which may be related to the fact that stachyose helps *B. megaterium* R32 multiply *in vivo* and maximize the probiotic effect of *Bacillus*. Therefore, stachyose and *B. megaterium* R32 can be used as potentially available substrates to improve the immunity and intestinal defense ability of turbot.

## Figures and Tables

**Table 1:** Formulation of spore solid medium.

Ingredients	Concentration (g/l)
Soy peptone	3.30
Beef extract	1.00
Nacl	5.00
Kcl	1.00
Dipotassium phosphate	2.00
Magnesium sulfate heptahydrate	0.25
Manganese sulfate	0.01
Lactose	5.00
Agar	15.00

**Table 2:** Formulation and proximate composition of the experimental diets (% dry matter).

Ingredients	C	RS	RC
Fish meal^1^	36.00	36.00	36.00
Soybean meal^1^	15.68	15.68	15.68
Corn protein flour	8.00	8.00	8.00
Gluten	5.12	5.12	5.12
Peanut meal	3.20	3.20	3.20
Beer yeast	2.50	2.50	2.50
Wheat flour	17.63	16.13	17.63
Taurine	1.00	1.00	1.00
Methionine^2^	0.26	0.26	0.26
Threonine^2^	0.18	0.18	0.18
Histidine^2^	0.19	0.19	0.19
Lysine^2^	0.74	0.74	0.74
Fish oil	7.00	7.00	7.00
Soy lecithin	1.00	1.00	1.00
Multivitamin mixture^3^	1.00	1.00	1.00
Choline chloride	0.25	0.25	0.25
Ethoxy quinoline	0.05	0.05	0.05
Calcium propionate	0.10	0.10	0.10
Diyttrium trioxide	0.10	0.10	0.10
Stachyose^4^	0.00	1.50	0.00
Total	100.00	100.00	100.00
Addition of *Bacillus* (CFU/g)	0.00	1 × 10^8^	1 × 10^8^
Feed nutritional composition (%/dry matter)	—	—	—
Crude protein	51.74	51.54	51.54
Crude lipid	13.70	14.55	14.55
Ash	8.93	8.93	8.93

^1^Fish meal: purchased from Qingdao Seven Great Bio-Tech Company Limited (Qingdao, China), crude protein: 72.28%, crude lipid: 10.62%; Soybean meal: purchased from Qingdao Seven Great Bio-Tech Company Limited (Qingdao, China), crude protein: 52.03%, crude lipid: 1.32%.

^2^Methionine, purity > 99%; Threonine, purity > 99%; Histidine, purity > 99%; Lysine, purity > 98%. Purchased from Shanghai Macklin Biochemical Co., Ltd. (Shanghai, China).

^3^Vitamin and mineral premix (providing for per kg diet): VA 232.2 mg, VD_3_ 3.75 mg, VE 6 g, VK3 1.2 g, VB1 900 mg, VB2 1.35 g, VB6 1.05 g, VB12 7.5 mg; Calcium pantothenate 4.5 g, VB3 6.75 g, VB9 375 mg, VB7 15 mg, VC 15 g, Inositol 10 g, FeSO_4_ 20 g, ZnSO_4_ 9.6 g, MnSO_4_ 5 g, CuSO_4_ 600 mg, CoCl_2_ 80 mg, Na_2_SeO_3_ 40 mg; Calcium iodate 80 mg. Purchased from Qingdao Master Bio-Tech Company Limited (Qingdao, China).

^4^Stachyose: effective content 80%. Gained from China National Research Institute of Food and Fermentation Industries (Beijing, China).

**Table 3:** Primers used in quantitative real-time PCR.

Target gene	Sequences of primers (5′−3′)	Annealing temp. (°C)	Accession number	Amplification efficiency (%)	Product length (bp)
*TNF-α*	F: GGACAGGGCTGGTACAACAC	58	AJ276709.1	98.30	87
	R: TTCAATTAGTGCCACGACAAAGAG				

*IFN-γ*	F: GCTTTCCCGATCATCTTCTG	58	DQ400686.1	95.90	102
	R: GGTTTCCCAGATTCCCATTC				

*TGF-β*	F: TCAGCATTCCAGATGTAGGTG	58	KU238187.1	97.10	115
	R: GGAGAGTGGCTTCAGTTTTTC				

*MLCK*	F: GACACGACTGGCACGCAGATC	58	XM_011621260.1	98.70	92
	R: CAGATGACTCCGATGCTCCACATG				

*IL-8*	F: CCTGCGGAGCCTCGGAGTG	58	AB125645.1	95.90	106
	R: TGACATCTTCAGAGTGGCAATGATCTC				

*Claudin-4*	F: ATGTGGAGTGTGTCGGCTT	58	MF370857.1	95.40	176
	R: AGACCTTGCACTGCATCTG				

*MUC-2*	F: GTTGGTGCAGCCGCATAG	58	KU238186.1	101.90	112
	R: CACTGGACGCTGGGAATG				

*MUC-5*	F: TTGTCCCTGACCAAGTGATG	58	JU370277.1	95.30	157
	R: ACAAAGCCTGTCCAAGATCG				

*Occludin*	F: ACTGGCATTCTTCATCGC	58	KU238182.1	95.70	189
	R: GGTACAGATTCTGGCACATC				

*IL-15*	F: GCTTTCCCGATCATCTTCTG	58	MG253620.1	96.30	97
	R: GGTTTCCCAGATTCCCATTC				

*IL-13*	F: GTGCCCAAAATGCGGAAAATAGT	58	KP985236.1	97.00	188
	R: GTTCATAGCCAGCGGGAAGACCT				

*JNK*	F: CAGCCAACAGACCAGACA	58	HS032063.1	101.50	142
	R: TGAAGCCCAGTAACATCG				

*P38*	F: GACGAACCCTGTTTCCTGGT	58	KU238181	101.10	174
	R: TCGGCTGCTGTTATTCGCTT				

*AP-1*	F: CAGCTGCGGCTTGAAGTTTT	58	XM_035612079	100.30	95
	R: GTTCTACGACGAAGCCGTGA				

*Cmyc*	F: TCGATCCATCTGTTGTTTTCCCG	58	AWP17105.1	101.30	125
	R: ACCGCTTCACTGCCTGTCTCTTC				

*β-Catenin*	F: GACGTGTGGTCAGCTGGCTGCGT	58	KT372083.1	103.80	134
	R: GTGGGCCTTGATCTGGGGGAATT				

*CyclinD1*	F: GTGCCCAAAATGCGGAAAATAGT	58	AWP02073.1	101.60	89
	R: GTTCATAGCCAGCGGGAAGACCT				

*GSK3β*	F: GACGTGTGGTCAGCTGGCTGCGT	58	KT372085.1	97.30	162
	R: GTGGGCCTTGATCTGGGGGAATT				

*Bax*	F: AGCATCTTTGCTGACGGGAT	58	MN782169	96.20	87
	R: GCGCTCTCTGATGACCTGAA				

*Caspase7*	F: CTCCATCCTGCAGCTCAACA	58	MF370858	103.90	122
	R: GGTGCACTTCATTCCCATGC				

*Caspase9*	F: CCCAGGACATGATCGACGAG	58	KY979512.1	96.70	114
	R: ACAATGGGAAGGCTCGACTG				

*Bid*	F: AGGGAGATCGGAGCCCAAAT	58	XM_035642534.2	99.30	92
	R: TCACTAGGGTGAGAGTCAGGG				

*Bcl-2*	F: CGGACAGAAGCAGTACCT	58	XM_035634237.2	102.40	107
	R: TAAGCCATTCTCTTCCACAG				

*MAPK*	F: CTCGTACCGCTGCGACAG	58	XM_035640562.1	106.20	153
	R: CAGAGGGCATCTTCACCAC				

*β-actin*	F: TCCCTTCTATCGTCGGTCGCCCC	58	AY008305.1	100.50	168
	R: TCTCCATGTCATCCCAGTTGGTC				

**Table 4:** Effects of stachyose and *Bacillus* on specific growth rate, feeding rate, and feed efficiency rate of turbots.

Groups	SGR (%/day)	FR (%/day)	FE	IW (g)	FW (g)	SR (%)
C	2.13 ± 0.07	1.56 ± 0.02	1.22 ± 0.04	12.04 ± 0.04	39.72 ± 1.40	100 ± 0.00
RS	2.02 ± 0.08	1.46 ± 0.08	1.26 ± 0.04	12.04 ± 0.01	37.81 ± 1.78	98.8 ± 0.70
RC	2.24 ± 0.07	1.63 ± 0.06	1.22 ± 0.04	11.89 ± 0.12	41.61 ± 1.50	100 ± 0.00

**Table 5:** PCoA between-group difference test statistics.

Adonis	Df	Sums_of_sqs	F.model	*R* ^2^	Pr (> *F*)
Group factors	2	1.1666	3.2752	0.3531	0.001
Residuals	12	2.1371	—	0.6469	0
Total	14	3.3037	—	1.0000	0

## Data Availability

All the data used to support the findings of this study are included in this paper.
